# Fenofibrate Enhances the *In Vitro* Differentiation of Foxp3^+^ Regulatory T Cells in Mice

**DOI:** 10.1155/2012/529035

**Published:** 2012-03-11

**Authors:** Zhou Zhou, Ying Liang, Yanxiang Gao, Wei Kong, Juan Feng, Xian Wang

**Affiliations:** Department of Physiology and Pathophysiology, School of Basic Medical Sciences, Peking University Health Science Center, Key Laboratory of Molecular Cardiovascular Science, Ministry of Education, Beijing 100191, China

## Abstract

Foxp3^+^ regulatory T cells (Tregs) play a critical role in maintaining immune self-tolerance. Reduced number and activity of Tregs are usually found in autoimmune and inflammatory diseases, and enhancing the differentiation of Tregs may be a promising therapeutic strategy. Some reports suggested an anti-inflammatory and anti-autoimmune potential for fenofibrate, a hypolipidemic drug used worldwide, whose lipid effects are mediated by the activation of peroxisome proliferator-activated receptor *α* (PPAR*α*). In the present paper, we found that fenofibrate dose-dependently increased transforming growth factor-*β* and interleukin-2-induced Treg differentiation *in vitro*, by 1.96-fold from 0 to 20 *μ*M (12.59 ± 1.34% to 24.69 ± 3.03%, *P* < 0.05). Other PPAR*α* activators, WY14643 (100 *μ*M), gemfibrozil (50 *μ*M), and bezafibrate (30 *μ*M), could not enhance Treg differentiation. In addition, PPAR*α* could not upregulate the promoter activity of the Treg-specific transcription factor Foxp3. Fenofibrate might exert its function by enhancing Smad3 phosphorylation, a critical signal in Treg differentiation, via Akt suppression. Our work reveals a new PPAR*α* independent anti-inflammatory mechanism of fenofibrate in up-regulating mouse Treg differentiation.

## 1. Introduction

The immune function is normally delicately regulated to maintain both host defense and self-tolerance. Apart from negative selection in the process of T-cell development, recently discovered Foxp3^+^ regulatory T cells (Tregs) represent another important aspect in preventing self-immune reaction [1]. Tregs are a unique group of T cells that mainly suppress immune reaction and inflammation caused by other immune cells [1]. The dysfunction and reduction in number of Tregs are found in many autoimmune and inflammatory diseases such as multiple sclerosis, inflammatory bowel diseases, type 1 diabetes, rheumatoid arthritis, systemic lupus erythematosus, psoriasis [2], and atherosclerosis [3]. Enhancing the Treg amount may be a promising therapeutic target for these diseases.

 Atherosclerosis is widely known as a chronic inflammatory disease with the malfunction of multiple subsets of immune cells [4, 5]. We and others have revealed a reduced Treg number involved in deteriorated atherosclerosis [3, 6]. As a widely used lipid-lowering anti-atherosclerosis drug [7], fenofibrate, a peroxisome proliferator-activated receptor *α* (PPAR*α*) activator, has been intensely studied. PPAR*α*-dependent and -independent anti-inflammatory activities were reported to play an important role in the function of fenofibrate, such as inhibiting NF-*κ*B activity in inflammation-related cells [7, 8]. As well, fenofibrate might modulate the differentiation of T helper type 1 (Th1) cells and Th2 cells [9, 10]. However, whether and how fenofibrate affects the differentiation of Tregs is still elusive.

 Differentiation is the core process in regulating Treg amount. Tregs can be differentiated from naïve T cells in the periphery with the induction of transforming growth factor-*β* (TGF-*β*) and interleukin-2 (IL-2) [11, 12]. Long term phosphorylation of Smad3, the downstream signal of TGF-*β*, is the key signal inducing the transcription factor Foxp3 [13], upon which the differentiation of Tregs commits [14]. Although several factors, such as Akt activation [15, 16], may inhibit Treg differentiation, clinical drugs targeting this process are still limited.

 In the present study, we found that fenofibrate improved the differentiation of Foxp3^+^ Treg cells induced with TGF-*β* and IL-2 *in vitro*, which might be associated with reduced Akt phosphorylation and enhanced long-term activation of Smad3. Other PPAR*α* activators, including bezafibrate, gemfibrozil, and WY14643, did not show the same activity as fenofibrate, so this Treg differentiation-improving function might be specific to fenofibrate and independent of PPAR*α* activation. 

## 2. Materials and Methods

### 2.1. Cell Sorting

 Six-week-old special pathogen-free female C57BL/6 mice were provided by the Animal Center of Peking University Health Science Center (Beijing, China). This study was carried out in accordance with the recommendations in the Guide for the Care and Use of Laboratory Animals of the Health Science Center of Peking University. The protocol was approved by the Committee on the Ethics of Animal Experiments of the Health Science Center of Peking University. All surgery was performed with mice under sodium pentobarbital anesthesia, and all efforts were made to minimize suffering. After mice were killed, total and CD4^+^ T cells of mouse spleens were enriched with positive selection magnetic microbeads against CD90.2 and CD4, respectively (Miltenyi Biotec, Bergisch Gladbach, Germany), following the manufacturer's instructions.

### 2.2. Cell Culture and Induction of Treg Differentiation *In Vitro*

Total and CD4^+^ T cells were purified and cultured as described [17] with minor modification. Briefly, 1 × 10^6^ total or CD4^+^ T cells were cultured with RPMI 1640 medium (Hyclone, Carlsbad, CA, USA) containing 10% fetal bovine serum (Hyclone) in each well of 48-well plates containing plate-bound anti-CD3 (1 *μ*g/mL, BD Pharmagen, Franklin Lakes, NJ, USA) and soluble anti-CD28 (1 *μ*g/mL, BD Pharmagen) antibodies. For Treg differentiation, cultures were supplemented with 5 ng/mL TGF-*β*1 (Pepro Tech, Rocky Hill, CT, USA) and 50 U/mL IL-2 (R&D Systems, Minneapolis, MN, USA). Anti-interferon- (IFN-) *γ* antibody (5 *μ*g/mL) and anti-IL-4 antibody (5 *μ*g/mL, both R&D Systems) were added as indicated. Fenofibrate, WY14643, bezafibrate, and gemfibrozil (all from Sigma Chemical, St. Louis, MO, USA) were used at the doses indicated. For flow cytometry and RT-PCR, cells were collected 4 days later. For western blot analysis, cells were collected 24 or 48 hours later.

### 2.3. Flow Cytometry

 For Foxp3 staining, cells were collected and stained with FITC or APC-tagged anti-Foxp3 antibody (eBioscience, San Diego, CA). For CD4, CD8, and Foxp3 multiple staining, cells were stained with FITC-tagged CD4 antibody and PE-tagged CD8 antibody (eBioscience), then with APC-tagged anti-Foxp3 antibody according to the manufacturer's instructions. Stained cells were analyzed by FACScan flow cytometry with Cell QuestPro software (BD Biosciences, USA).

### 2.4. Real-Time RT-PCR Analysis

 Cells were collected 4 days after the initiation of Treg cell differentiation. RNA isolation and real-time RT-PCR were performed as described [18]. Briefly, total RNA was extracted by the TRIzol reagent method (Invitrogen, Carlsbad, CA, USA). One microgram of total RNA per sample was reverse-transcribed with use of the AMV Reverse Transcription System (Promega, Madison, WI, USA). Real-time PCR amplifications involved an Mx3000 Multiplex Quantitative PCR System (Stratagene Corp, La Jolla, CA, USA) and SYBR Green I reagent.

All amplification reactions were carried out for 40 cycles (an initial stage of 7 min at 95°C, followed by a three-step cycle of 20 s at 94°C, 25 s at 60°C, and 30 s at 72°C) and were performed in duplicate. The accuracy of PCR products was confirmed by sequencing amplicons. The relative target mRNA levels were assessed with use of Stratagene Mx3000 software and normalized to that of the internal control, *β*-actin. The primers were for Foxp3, forward, TCCTTCCCAGAGTTCTTCCAC and reverse, ACTTGTGCAGGCTCAGGTTGT; *β*-actin, forward, ATCTGGCACCACACCTTC and reverse, AGCCAGGTCCAGACGCA.

### 2.5. Western Blot Analysis

 Immunoblotting was performed as described [18]. Briefly, T cell lysis samples containing the same amount of protein were resolved on 10% SDS-PAGE. The membranes were incubated with primary antibodies and then IRDye 800DX- or IRDye 700DX-conjugated secondary antibodies (Rockland, Gilbertsville, PA, USA). The immunofluorescence signal was detected by use of the Odyssey infrared imaging system (LICOR Biosciences, Lincoln, NB, USA). The primary antibodies included anti-phosphorylated-STAT5, anti-total and phosphorylated-Akt, anti-total-Smad3, anti-*β*-actin antibodies (all from Cell Signal Technology, Danvers, MA, USA), anti-phosphorylated-Smad3 antibody (Millipore, Billerica, MA, USA), anti-total-STAT5, and anti-eIF5 antibodies (both from Santa Cruz Biotechnology, CA, USA).

### 2.6. Transfection and Luciferase Reporter Assay

Transfection and luciferase reporter assays were performed as described [19] with minor modification. Briefly, mouse embryonic fibroblast cells or HEK 293A cells were transfected with 0.1 *μ*g Foxp3 promoter reporter plasmid or 0.1 *μ*g peroxisome proliferator response element (PPRE) luciferase reporter plasmid, together with *β*-galactosidase-expressing plasmid as an internal reference with use of cationic polymer transfection reagent (JetPEI, France), as well as 0.2 *μ*g PPAR*α* plasmid if indicated, or 0.2 *μ*g control plasmid GFP. After transfection for 4 hours, the cells were incubated with fresh Dulbecco modified Eagle medium (DMEM) containing 10% fetal bovine serum and fenofibrate, gemfibrozil, WY14643, bezafibrate, phorbol 12-myristate 13-acetate (PMA) plus ionomycin, or solute control, respectively. 24 hours later, the growth medium was removed and replaced with 200 *μ*L of reporter lysis buffer and the luciferase and *β*-galactosidase activity was measured with use of a luciferase assay system (Promega, Madison, WI, USA).

### 2.7. Statistical Analysis

 All data are expressed as mean ± SEM or original data representing 1 of at least 3 independent experiments. Unpaired Student *t* test was used to compare 2 groups and one-way ANOVA followed by Newman-Keul post-hoc test to compare multiple groups. *P* < 0.05 was considered statistically significant.

## 3. Results

### 3.1. Fenofibrate Enhanced the Differentiation of Tregs *In Vitro*

**Figure 1:**
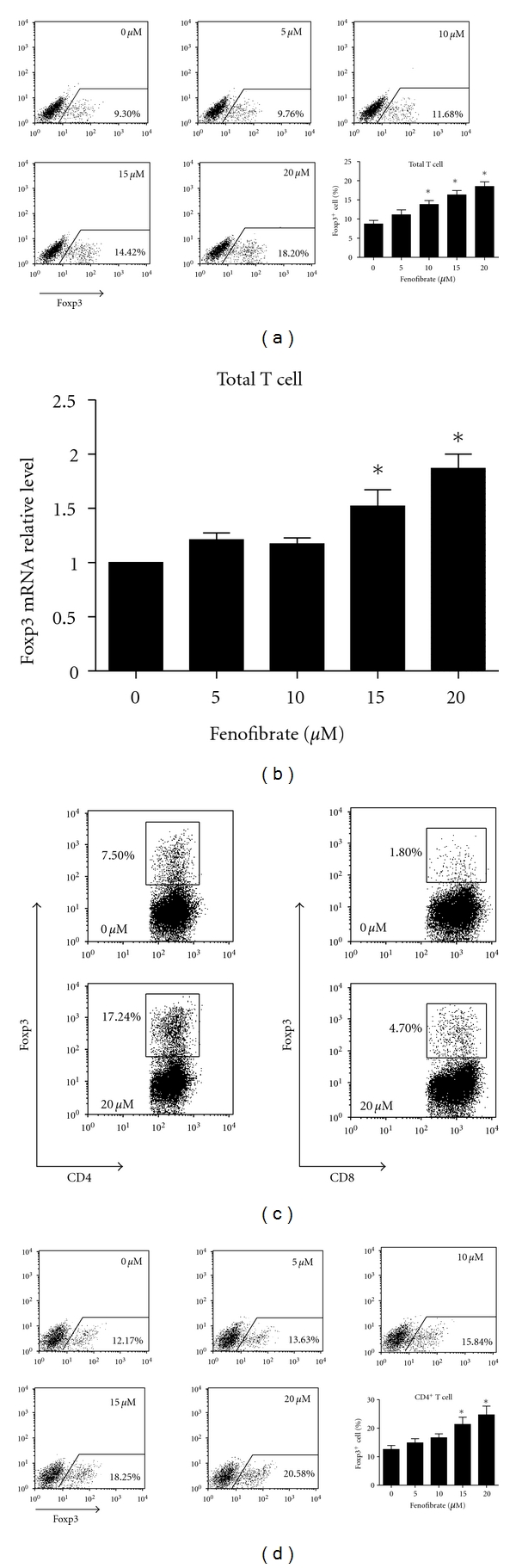
Fenofibrate promoted the differentiation of Tregs* in vitro*. Total T cells (a) or CD4^+  ^T cells (d) were isolated from mouse spleens and induced to differentiate into Tregs with 5 ng/mL TGF-*β*, 50 U/mL IL-2, and fenofibrate doses indicated. The percentage of Foxp3^+^ T cells was analyzed by flow cytometry 4 days later. The mRNA level of Foxp3 in total T cells was analyzed by real-time PCR (b), and the percentages of Foxp3^+^ T cells in CD4^+^ and CD8^+^ T cell subgroups were analyzed by flow cytometry (c). In (a) and (d), numbers in the upper right corner indicate the final concentration of fenofibrate, and the outlined areas indicate Foxp3^+^ T cells with percentages shown in the lower right corner, *n* = 5~6, *P < 0.05 versus 0 *μ*M group. In (b) *n* = 4, *P < 0.05  versus 0 *μ*M group. In (c) data represent 1 of at least 3 independent experiments.

 To determine the effect of fenofibrate on the differentiation of Tregs, we induced Treg differentiation *in vitro* with TGF-*β* and IL-2. Fenofibrate (10~20 *μ*M) dose-dependently potentiated the ratio of differentiated Foxp3^+^ T cells to total T cells after 4-day induction. An amount of 20 *μ*M fenofibrate elevated the Foxp3^+^ cell percentage by 2.12-fold, from 8.72 ± 0.95% to 18.51 ± 1.21% (*P* < 0.05) (Figure 1(a)). The mRNA level of Foxp3 was increased accordant with enhanced Treg differentiation by fenofibrate (Figure 1(b)). The live cell ratio was approximately 70% in all the groups (data not shown). CD4^+^ and CD8^+^ T cells were both induced to express Foxp3 by TGF-*β* and IL-2, with most being CD4^+^ T cells. Fenofibrate elevated the percentage of Foxp3^+^ cells from both cell subgroups (Figure 1(c)), which suggests that the effect of fenofibrate is not restricted to CD4^+^ or CD8^+^ T cells. In addition, Treg differentiation from purified CD4^+^ T cells was also improved dose-dependently by fenofibrate. Fenofibrate promoted the Treg differentiation 1.96-fold from 12.59 ± 1.34% to 24.69 ± 3.03% at 20 *μ*M (*P* < 0.05) in CD4^+^ T cells (Figure 1(d)). These data suggest that fenofibrate improves the differentiation of Treg cells *in vitro*. 

### 3.2. IFN-*γ* and IL-4 Were Not Involved in the Effect of Fenofibrate on Treg Differentiation

**Figure 2:**
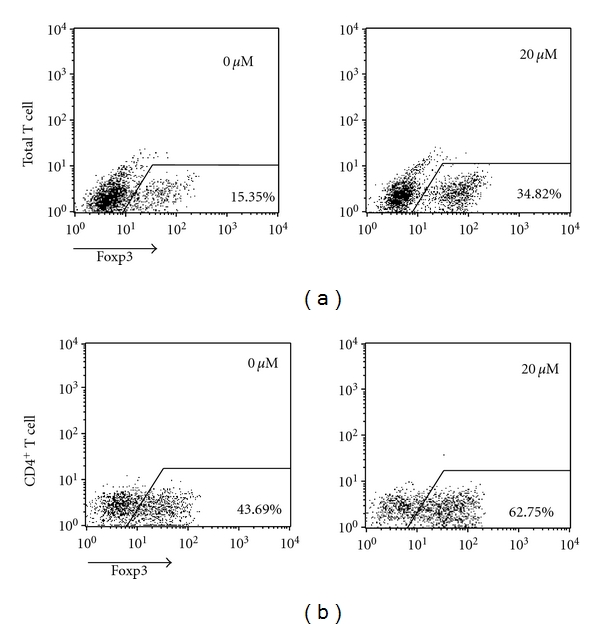
The effect of fenofibrate on Tregs did not involve changes in IFN-*γ* and IL-4 expression. Total T cells (a) and CD4^+^ T cells (b) were isolated from mouse spleens and induced to differentiate into Tregs with 5 ng/mL TGF-*β*, 50 U/mL IL-2, 5 *μ*g/mL IFN-*γ* neutralizing antibody, 5 *μ*g/mL IL-4 neutralizing antibody, and fenofibrate doses indicated. After 4 days of induction, the percentage of Foxp3^+^ T cells was analyzed by flow cytometry. Numbers in the upper right corner indicate the final concentration of fenofibrate, and the outlined areas indicate Foxp3^+^ T cells with percentages shown in the lower right corner. Data represent 1 of at least 3 independent experiments.

Previous studies reported that fenofibrate could reduce the expression of IFN-*γ* and enhance the expression of IL-4 from activated T cells [9, 10]. Because these 2 cytokines were both shown to inhibit Treg differentiation, we examined whether fenofibrate exerted its function by interfering with the secretion of IFN-*γ* and IL-4. Anti-IFN-*γ* and anti-IL-4 neutralizing antibodies were supplemented in the Treg differentiation system, but fenofibrate could still up-regulate the rate of differentiated Foxp3^+^ T cells from total T cells and purified CD4^+^ T cells (Figure 2). Therefore, fenofibrate enhanced Treg differentiation independent of IFN-*γ* or IL-4.

### 3.3. PPAR*α* Activation Was Not Involved in the Function of Fenofibrate

**Figure 3:**
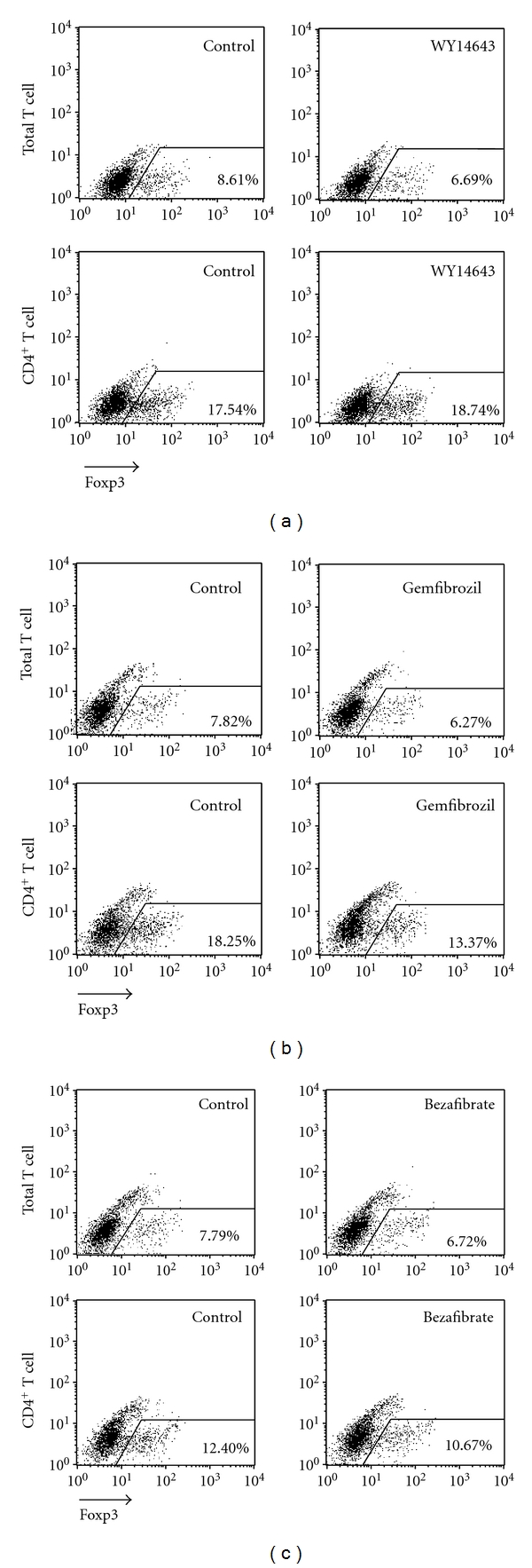
WY14643, gemfibrozil, and bezafibrate did not affect Treg differentiation *in vitro*. Total and CD4^+^ T cells were isolated from mouse spleens and induced to differentiate into Treg cells with 5 ng/mL TGF-*β* and 50 U/mL IL-2. 100 *μ*M WY14643 (a), 50 *μ*M gemfibrozil (b) and 30 *μ*M bezafibrate (c) or solute control were added respectively. 4 days later the percentage of Foxp3^+^ T cells was analyzed with flow cytometry. The outlined areas indicate Foxp3^+^ T cells, and the percentages were shown in the lower right corner. Data represent 1 of at least 3 independent experiments.

 Because fenofibrate is well known as a recombinant PPAR*α* activator, we next checked whether fenofibrate exerted its Treg-differentiation stimulating function through PPAR*α*. First, we examined whether fenofibrate shared a similar function with other PPAR*α* activators. Three other PPAR*α* activators, 100 *μ*M WY14643, 50 *μ*M gemfibrozil, and 30 *μ*M bezafibrate (Figures 3(a), 3(b), and 3(c), resp.) were added to the Treg differentiation system, but none of them up-regulated the differentiation of Treg cells induced with TGF-*β* and IL-2.

**Figure 4:**
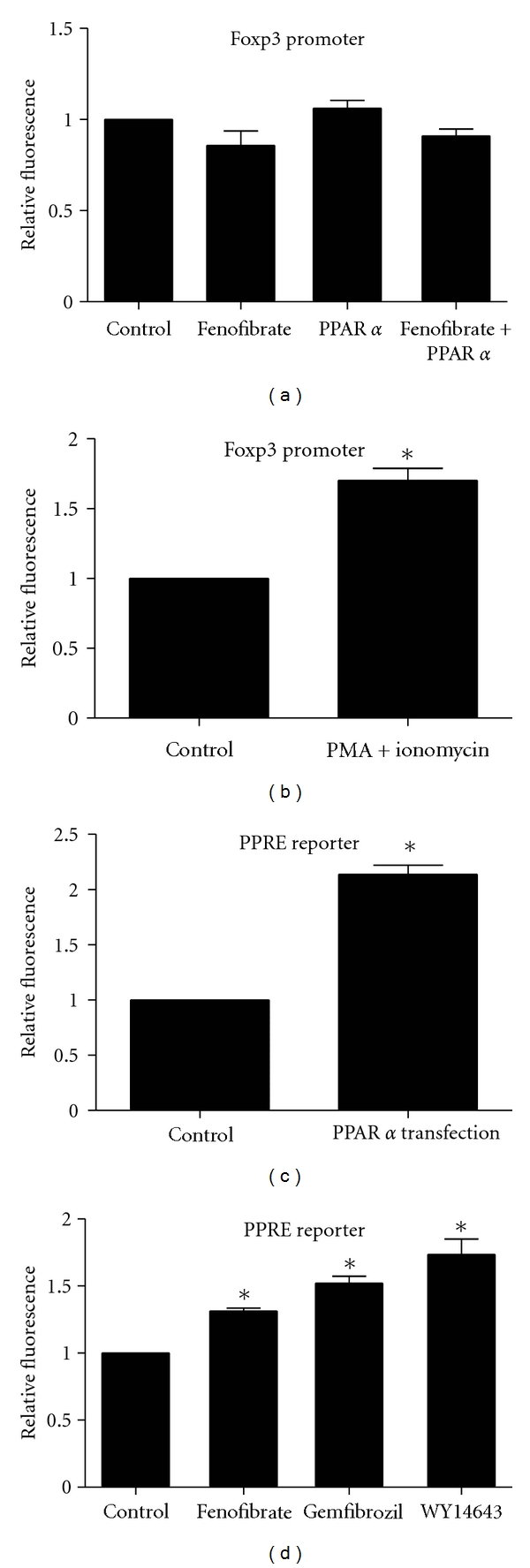
PPAR*α* activation could not improve Foxp3 promoter activity. (a) MEF cells were transfected with Foxp3 promoter-luciferase reporter plasmid along with *β*-galactosidase plasmid, then stimulated with 20 *μ*M fenofibrate or further transfected with PPAR*α* plasmid. All groups received equal amount of solute and plasmid adjusted with ethanol and GFP plasmid. (b) Foxp3 promoter-luciferase reporter plasmid and *β*-galactosidase plasmid were transfected into MEF cells and then the cells were stimulated with 50 ng/mL PMA and 1 *μ*g/mL ionomycin. PPRE-luciferase reporter plasmid and *β*-galactosidase plasmid were transfected to MEF cells with PPAR*α* expression plasmid or equal amount of GFP control plasmid (c) or to HEK 293 cells that were immediately stimulated with 20 *μ*M fenofibrate, 50 *μ*M gemfibrozil or 100 *μ*M WY14643 (d). 24 hours later, luciferase activity relative to *β*-galactosidase activity was analyzed. *n* = 5, *P < 0.05.

To further examine whether PPAR*α* activation modulated the expression of Foxp3, we tested whether the promoter activity of Foxp3 could be improved by fenofibrate or PPAR*α* over-expression. We constructed the luciferase reporter of Foxp3 promoter and transfected it into mouse embryonic fibroblast (MEF) cells. The luciferase activity was not affected by the stimulation of 20 *μ*M fenofibrate or further expression of PPAR*α* (Figure 4(a)). The efficacy of Foxp3 promoter was tested by the stimulating effect of 50 ng/mL PMA and 1 *μ*g/mL ionomycin (Figure 4(b)). The efficiency of PPAR*α* expression and activation was confirmed by the activation of the PPRE luciferase reporter (Figures 4(c), 4(d), and, see supplemental figure, in supplementary material available online at doi: 10.1155/2012/529035). These data suggest that Foxp3 promoter activity could not be influenced by PPAR*α*, and PPAR*α* activation might not be involved in fenofibrate-enhanced Treg differentiation.

### 3.4. Fenofibrate Improved the Phosphorylation of Smad3

**Figure 5:**
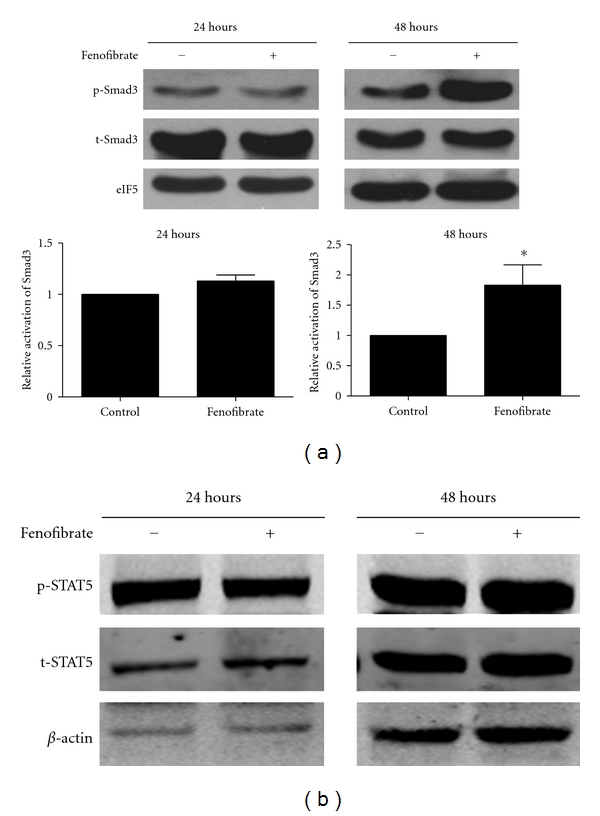
Fenofibrate enhanced Smad3 activation. Total mouse splenic T cells were induced to differentiate into Treg cells by use of 5 ng/mL TGF-*β* and 50 U/mL IL-2, and 20 *μ*M fenofibrate or solute control. 24 and 48 hours later, the phosphorylation of Smad3 (a) and STAT5 (b) as well as the total protein level were examined by western blot analysis. In (a) *n* = 6, *P < 0.05 versus solute control group. In (b) data represent 1 of at least 3 independent experiments.

 We next investigated the mechanism of the fenofibrate effect on Treg differentiation. TGF-*β* and IL-2 are 2 major cytokines that induce the differentiation of Tregs [17], so we checked whether fenofibrate regulated their signals. Long term activation of Smad3, downstream of TGF-*β*, is a critical signal in enhancing the differentiation of Tregs [20, 21]. In our study, phosphorylation of Smad3 was significantly up-regulated by fenofibrate 48 hours after the induction, with only slight effect at 24 hours (Figure 5(a)). However, phosphorylation of STAT5, the downstream signal of IL-2, was not affected by fenofibrate (Figure 5(b)). These data indicate that enhancing the TGF-*β* signal might be a critical step in improving Treg differentiation by fenofibrate, and the IL-2 signal might not take a major part.

### 3.5. Fenofibrate Suppressed the Activation of Akt

**Figure 6:**
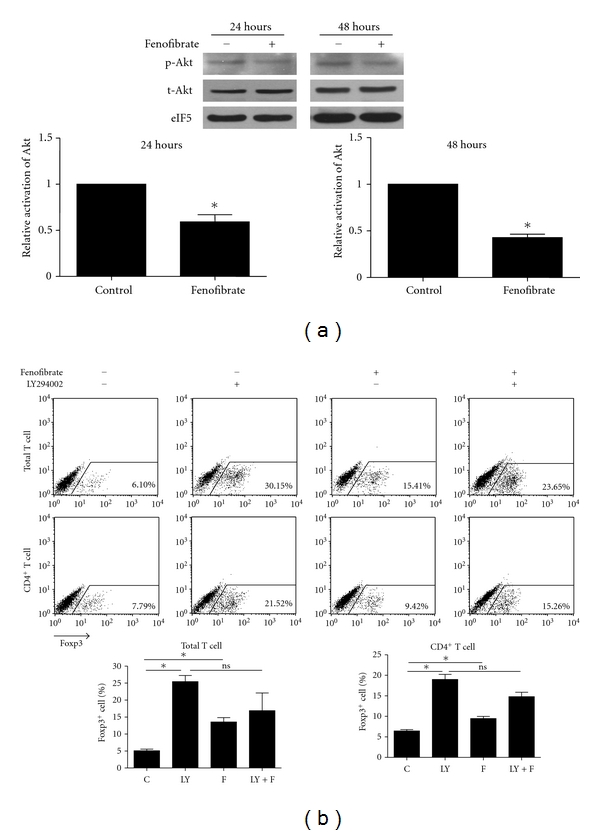
Fenofibrate reduced Akt phosphorylation. (a) Total mouse splenic T cells were induced to differentiate into Treg cells with 5 ng/mL TGF-*β* and 50 U/mL IL-2, and 20 *μ*M fenofibrate or solute control. 24 hours and 48 hours later, the activation of Akt and the total protein level were tested by western blot analysis. (b) Total or CD4^+^ mouse splenic T cells were induced with 5 ng/mL TGF-*β* and 50 U/mL IL-2 to differentiate into Treg cells with 1 *μ*M LY294002 (LY) and 20 *μ*M fenofibrate (F) as indicated in the figure. 4 days later the percentages of Foxp3^+^ T cells were analyzed with flow cytometry. In both (a) and (b), *n* = 3, *P < 0.05 versus solute control group.

 Akt activation is an important inhibitory signal for Treg differentiation [15, 16]. As well, activated Akt can block the phosphorylation of Smad3 by the TGF-*β* receptor complex [22]. We found Akt activation significantly inhibited by fenofibrate 24 hours and 48 hours after Treg induction (Figure 6(a)). Furthermore, an Akt signal blocker, LY294002, sufficiently improved Treg differentiation, confirming the inhibitory effect of Akt signal on Treg differentiation. Moreover, in the presence of LY294002, fenofibrate could not further enhance Treg differentiation (Figure 6(b)). Thus, fenofibrate probably enhanced Smad3 activation and then Treg differentiation at least in part by suppressing the phosphorylation of Akt, which was important in its very function.

## 4. Discussion

Regulatory T cells are a recently discovered T cell subgroup that plays a critical role in maintaining immune self-tolerance and controlling immune activity [1]. Modulating the differentiation of Treg cells may be a promising therapeutic target for treating autoimmune and inflammatory diseases [2]. However, artificial exogenous chemical compounds improving Treg differentiation are still limited. In the present study, we found that a hypolipidemic drug, fenofibrate, enhanced the differentiation of Tregs induced by TGF-*β* and IL-2 *in vitro*, probably by inhibiting Akt activation and then enhancing the TGF-*β* signal, Smad3 phosphorylation. Our study might provide information on a new promising drug for treating autoimmune and inflammatory diseases.

Retinoic acid [23] and 2,3,7,8-tetrachlorodibenzo-p-dioxin [24] were previously found to improve the differentiation of Treg cells *in vivo* and *in vitro*. Both of these chemicals are ligands of nuclear receptors, retinoic acid receptor and aryl hydrocarbon receptor, respectively, and their activation of the receptors was key to improving Treg differentiation [23, 24]. However, we found that fenofibrate, as a chemical ligand of nuclear receptor PPAR*α* [7], exerted its effect on Treg differentiation probably independent of PPAR*α* activation. We and others showed that PPAR*α* activation could not enhance the promoter activity of the Treg-specific transcription factor Foxp3 [25]. Also, other PPAR*α* activators, WY14643, gemfibrozil, and bezafibrate, could not affect Treg differentiation. So fenofibrate might be a unique member of the PPAR*α* ligands in regulating Treg differentiation.

Long-term Smad3 activation is a critical signal inducing the expression of Foxp3 and then Treg differentiation. The positive effect of retinoic acid [20] and S1P_1_ blockers [21] on the differentiation of Treg cells were both at least in part due to enhancing long term Smad3 phosphorylation. In our study, we for the first time in the world revealed that fenofibrate enhanced Smad3 activation by TGF-*β*, which might explain its effect on Treg differentiation. In contrast to Smad3, Akt activation is an important endogenous suppressive signal for Treg differentiation [15, 16]. Although the inhibitory effect of fenofibrate on Akt activation was recently found outside the immune system [26], we have supplied the first evidence that fenofibrate could also suppress Akt phosphorylation in T cells. Activated Akt can block the signal transduction from the activated TGF-*β* receptor complex to Smad3 at the molecular level [22, 27]. Moreover, low level Akt phosphorylation and high level Smad3 phosphorylation were also found in the same Treg favoring conditions [21]. So, inhibition of Akt might be an upstream signal of fenofibrate enhancing Smad3 activation. This Akt, Smad3, Foxp3 cascade might explain at least in part the positive effect of fenofibrate on Treg differentiation. Also, this Akt suppressing effect might improve Treg differentiation via other mechanisms independent of Smad3 enhancement.

Besides the peripheral differentiation induced by TGF-*β* and IL-2, Treg cells can also be directly induced in the thymus [28]. Whether this thymus Treg differentiation is modulated by fenofibrate and the overall action of the immune system with fenofibrate-enhanced Treg differentiation improves autoimmune and inflammatory diseases *in vivo* remain for further investigations.

 Autoimmune and inflammatory diseases are severe diseases for humans, and the current treatments are limited. We for the first time revealed that fenofibrate could improve Treg differentiation *in vitro*, with implications for ameliorating autoimmune and inflammatory diseases. To our knowledge, fenofibrate is the first widely used drug to possess this function. Added to its previously found anti-inflammatory and Th1 differentiation functions, fenofibrate might be a safe alternative for treating autoimmune and inflammatory diseases.
